# *Q*-Factor Optimization of Modes in Ordered and Disordered Photonic Systems Using Non-Hermitian Perturbation Theory

**DOI:** 10.1021/acsphotonics.3c00510

**Published:** 2023-07-10

**Authors:** Nicoletta Granchi, Francesca Intonti, Marian Florescu, Pedro David García, Massimo Gurioli, Guillermo Arregui

**Affiliations:** † Department of Physics, University of Florence, via Sansone 1, I-50019 Sesto Fiorentino, FI, Italy; ‡ European Laboratory for Nonlinear Spectroscopy, via Nello Carrara 1, I-50019 Sesto Fiorentino, FI, Italy; § Advanced Technology Institute and Department of Physics, University of Surrey, Guildford, Surrey GU2 7XH, U.K.; ∥ Instituto de Ciencia de Materiales de Madrid (ICMM), Consejo Superior de Investigaciones Científicas (CSIC), Calle Sor Juana Inés de la Cruz 3, 28049 Madrid, Spain; ⊥ Department of Electrical and Photonics Engineering, DTU Electro, Technical University of Denmark, Building 343, DK-2800 Kgs. Lyngby, Denmark

**Keywords:** photonic resonators, quasinormal modes, Q-factor optimization, non-Hermitian perturbation theory, random systems, Anderson modes

## Abstract

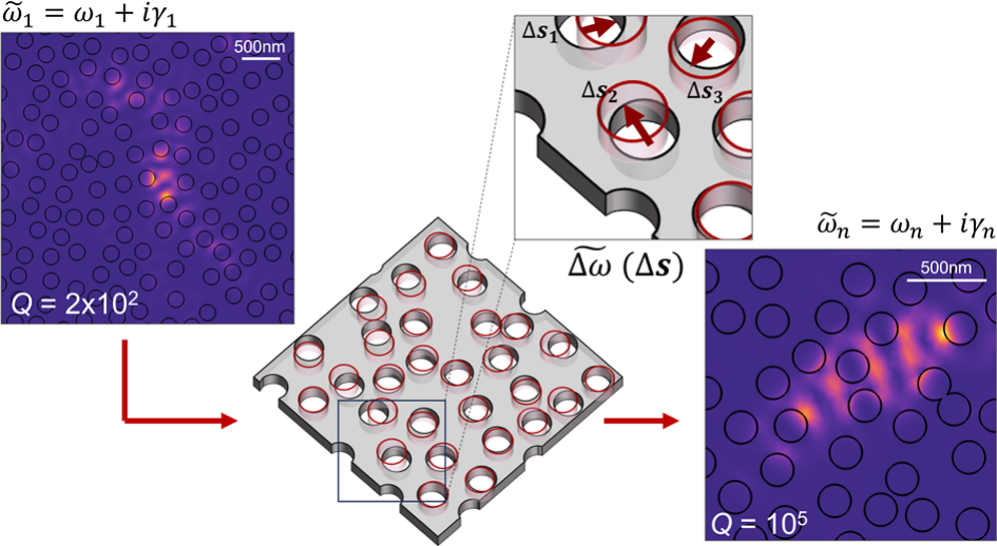

The quality factor, *Q*, of photonic resonators permeates most figures of merit in applications that rely on cavity-enhanced light–matter interaction such as all-optical information processing, high-resolution sensing, or ultralow-threshold lasing. As a consequence, large-scale efforts have been devoted to understanding and efficiently computing and optimizing the *Q* of optical resonators in the design stage. This has generated large know-how on the relation between physical quantities of the cavity, e.g., *Q*, and controllable parameters, e.g., hole positions, for engineered cavities in gaped photonic crystals. However, such a correspondence is much less intuitive in the case of modes in disordered photonic media, e.g., Anderson-localized modes. Here, we demonstrate that the theoretical framework of quasinormal modes (QNMs), a non-Hermitian perturbation theory for shifting material boundaries, and a finite-element complex eigensolver provide an ideal toolbox for the automated shape optimization of *Q* of a single photonic mode in both ordered and disordered environments. We benchmark the non-Hermitian perturbation formula and employ it to optimize the Q-factor of a photonic mode relative to the position of vertically etched holes in a dielectric slab for two different settings: first, for the fundamental mode of L3 cavities with various footprints, demonstrating that the approach simultaneously takes in-plane and out-of-plane losses into account and leads to minor modal structure modifications; and second, for an Anderson-localized mode with an initial *Q* of 200, which evolves into a completely different mode, displaying a threefold reduction in the mode volume, a different overall spatial location, and, notably, a 3 order of magnitude increase in *Q*.

## Introduction

The interaction of light and matter in structured optical environments that tailor the local density of optical states is at the core of fields such as cavity electrodynamics,^1−3^ nonlinear optics,^4−6^ and optomechanics.^7,8^ In many of these fields, the use of photonic crystals, their band gaps, and engineered defects within them, such as cavities and waveguides, is widespread.^9^ However, the translational order that underpins such synthetic materials is not necessary, and disordered systems can expand the parameter space for several applications due to the large plethora of design freedom. Moreover, disordered photonic media made of random distributions of pointlike scatterers with controlled scattering properties have also been shown to block, guide, and tightly confine light.^10−13^ In addition, the nontrivial interplay of order and disorder can also drastically reshape light transport, with strong Anderson localization of light as an emblematic example.^14^ This has fostered the vision of a vast landscape from order to disorder with engineered disordered systems as a complementary alternative to their fully ordered counterpart.^15^ While the mechanisms governing light transport in ordered and disordered environments may differ, their fitness as light–matter interfaces is ultimately determined by their ability to sustain photonic modes with large optical energy densities, i.e., through spectral and spatial light confinement. A paradigmatic way of doing so^16^ is via high quality factor, *Q*, and low mode volume, *V*, optical cavities, with the latter figure of merit taking a different expression depending on the interaction at hand.^17^ Given the generalized role of *Q*,^18^ extensive efforts have been put into improving the designs and top-down nanofabrication. While enhancements of various orders of magnitude in *Q* can be achieved through intuitive-based approaches^19^ and radiation-limited *Q*s as high as 9 million have been demonstrated in optimized two-dimensional photonic-crystal cavities,^20^ progress in the case of random photonic systems has been more limited.^21^ Such an issue has been addressed at the ensemble-average level by introducing short-range correlations,^22−24^ but the *Q*s of Anderson-localized modes are only on par with engineered cavities in the case of slow-light photonic-crystal waveguides subjected to minute fabrication disorder.^25^ On the other hand, the alternative problem of optimizing the *Q* of a single localized photonic mode in a random system, i.e., to engineer it, has not been tackled. In the more general picture of wave-matter science, while the optimization of ordered systems can be considered unambiguous, engineering and optimizing performances of single realizations of disordered systems is more difficult. Several approaches have tackled this challenge, for example, connecting wave-physics to network science, and succeeded in establishing clear interplays between physical quantities and tunable parameters.^26−28^

In the absence of absorption, the *Q* of a cavity mode is determined by radiation losses at the *boundaries* of the domain. Due to its compatibility with conventional planar semiconductor technology, the preferred geometry is a dielectric slab: this leads to a heuristic distinction between in-plane and out-of-plane losses, respectively, gauged by *Q*_∥_ and *Q*_⊥_. The possibility of increasing the former by increasing the footprint in the slab plane has implied that most efforts to maximize *Q* have been devoted to maximizing *Q*_⊥_. This boils down to modifying the momentum-space representation of the resonant modes via either first-principles group symmetry arguments,^29^ the direct observation of the smoothness of the field envelope,^30^ real-space analysis of the leaky components,^31^ or semianalytic formalisms that tackle the problem as a reverse design one.^32,33^ However, while they allow a pathway for iterative optimization, these approaches are supervised, and their extension to the case of random modes is not trivial. In parallel, rapid growth of computational resources has helped the development of both gradient-free and gradient-based automated optimization methods such as nature-inspired search algorithms,^34,35^ machine learning,^27,36,37^ and density-based topology optimization.^38^ In particular, gradient-based inverse design, which is transforming the paradigm of high-efficiency component design in nanophotonics,^39^ uses adjoint sensitivity analysis to efficiently compute gradients of a wide variety of objective functions. Traditionally used in finite difference and finite element solvers,^40,41^ the adjoint method has recently been extended to mode-expansion solvers through automatic differentiation techniques.^42^ Among the many desired functional characteristics, these methods have been employed to optimize the *Q* of a photonic mode.^42^ We note, however, that these have rarely relied on directly solving Maxwell’s eigenproblem with radiation boundary conditions,^43^ where *Q* emerges as a natural quantity through the complex eigenfrequencies of quasinormal modes (QNMs).^44^ Here, we propose a gradient-based automated optimization approach to maximize the *Q* of optical resonances in ordered and disordered dielectric slabs. The method uses first-order non-Hermitian perturbation theory^45^ to efficiently compute the gradients of the *Q*-factor of a single QNM relative to arbitrary material boundary displacements, i.e., it optimizes the position and shape of material boundaries. First, we exploit the method on L3 cavities surrounded by photonic crystals of different spatial extensions, i.e., of different footprints, and evidence how it naturally optimizes for both *Q*_⊥_ and *Q*_∥_. Then, we employ it to optimize the *Q* of an Anderson mode supported by a dielectric slab with a random distribution of etched holes^46^ and demonstrate the optimization process to produce a 3 order of magnitude enhancement of its *Q*. By monitoring the spatial distribution of the mode along the optimization, we observe the central location and spatial distribution of the mode to change dramatically, with a final spatial localization comparable to the one achieved in engineered photonic-crystal cavities.

## Q-Factor Optimization Method

**Figure 1:**
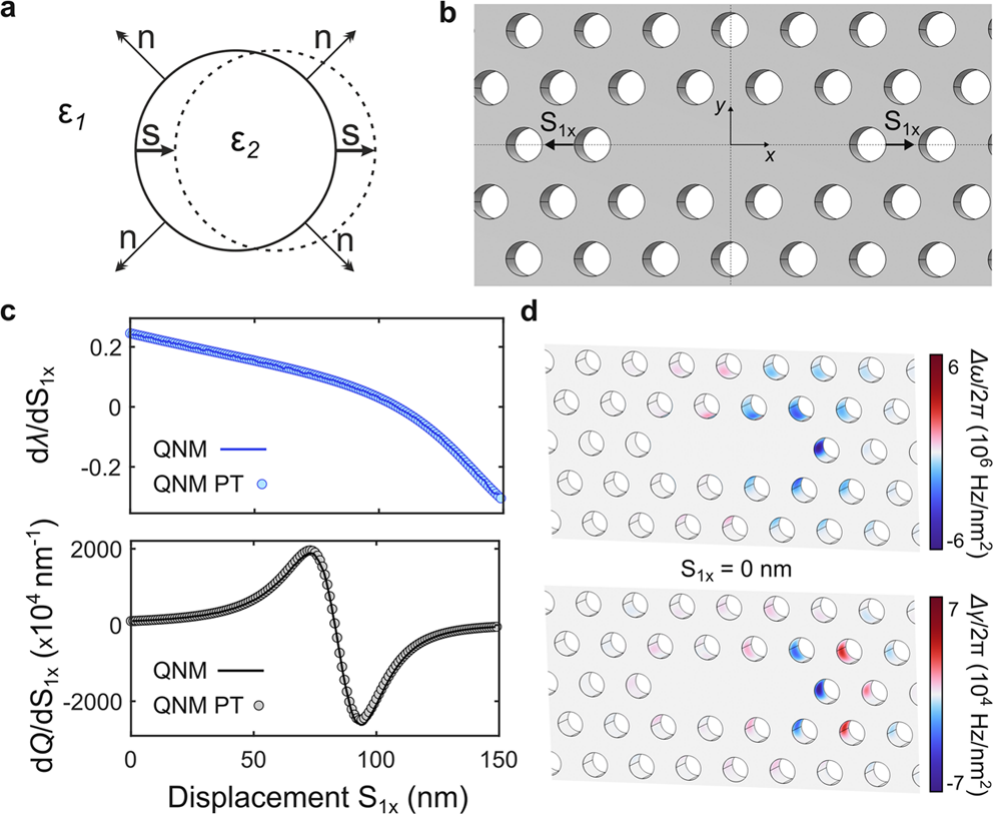
Quasinormal mode (QNM) perturbation theory on resonators with material boundary shifts. (a) Sketch of the displacement *S⃗* of a single hole of dielectric constant ε_2_ in a medium with dielectric constant ε_1_. (b) Geometry of an L3 cavity with lattice constant *a* = 420 nm, air hole radius *r* = 0.265*a*, slab thickness *d* = 220 nm, and refractive index *n* = 3.46. The total system size is set to *L*_*x*_ = 20*a* and *L*_*y*_ = 20*a*√3/2, which leads to *N* = 200 holes in total. The displacement in the *x* direction of the first holes in the cavity axis, *S*_1*x*_, is represented, as well as the symmetry employed in the simulation. (c) Derivatives of the resonant wavelength and quality factor *Q* of the L3 cavity relative to *S*_1*x*_ as a function of *S*_1*x*_. Both values calculated through the QNM eigenfrequency ω̃ from the numerical solver (line) and obtained from the perturbation theory (circles) are included. (d) Real and imaginary parts of the integrand of eq 1 for s = (*S*_*ix*_, 0), with *i* being the index for the *i*-th hole.

Resonant electromagnetic fields in plasmonic and dielectric resonators are unbound; this gives rise to, e.g., an exponential decay of the resonating field after an excitation is switched off or lineshapes of finite linewidth in scattering spectra. From a modeling perspective, these resonances are well described within the theoretical framework of QNMs, which are the solutions to the source-free Maxwell wave equation with a radiation boundary condition.^44,45^ The resulting eigenvalue problem admits solutions with complex eigenfrequencies ω̃_*n*_ = ω_*n*_ + *i*γ_*n*_, from where the *Q*-factor of the *n*-th mode is found as *Q*_*n*_ = ω_*n*_/2γ_*n*_. As a consequence of the radiation condition, the QNM fields diverge in the far field, which invalidates common energy normalization approaches in Hermitian systems. This is circumvented through alternative normalization approaches that regularize the QNM behavior.^47^ In this work, we use the so-called perfectly matched layer (PML) normalization^48^
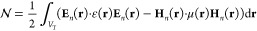
1where {**E**_*n*_, **H**_*n*_} is the electromagnetic field of the QNM and the integral is carried out over the volume *V*_T_ = *V* ∪ *V*_PML_, which includes the volume surrounding the cavity, *V*, and importantly, the volume *V*_PML_ occupied by the PML used for the numerical implementation of the radiation condition. In recent years, various QNM expansion techniques have been used to model light-scattering problems^49,50^ and light–matter interaction^48,51−53^ whenever either (or both) photonic or (and) plasmonic resonances are involved. In addition, perturbation theories have been adequately generalized to open resonators using QNMs^54^ and their predictions experimentally tested.^55,56^ Here, we consider the effect of shifting the boundaries between two materials (labeled 1 and 2). The first-order complex shift to the complex eigenfrequency ω̃_*n*_ of a QNM is given by^45,58^
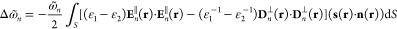
2where **E**_*n*_ and **D**_*n*_ are the normalized (according to eq 1) complex electric and displacement fields of the QNM, respectively; the superscripts “∥” and “⊥” denote field components, respectively, parallel and perpendicular to the shifted boundary *S*, the displacement of which is given by **s**(**r**) and its normal by **n**(**r**) pointing from material 1 to material 2 (see Figure 1a). The expression in eq 2 generalizes the formula in^57^ to open resonators and has been recently employed to calculate dissipative optomechanical coupling rates,^58^ the sensitivity of ultra-low mode volume dielectric bowtie nanocavities,^59^ and the effect of surface roughness in plasmonic resonators.^60^ Even if the use of the QNM perturbation theory for shape deformations has been proposed to optimize *Q*,^61^ a systematic study evidencing such use is still missing.

In this work, we study the photonic modes of dielectric slabs with *n* vertically etched void features, an example of which is an L3 photonic-crystal cavity,^19^ whose geometry is shown in Figure 1b. We validate eq 2 by computing the QNM associated with the fundamental mode (the so-called Y mode) of an L3 cavity as a function of a symmetric and rigid shift **s** = (*S*_1*x*_,0) in the position of the two holes bounding the cavity along its axis. We use a commercial finite-element complex eigensolver (COMSOL Multiphysics^62^) and reduce the computational size by employing the appropriate boundary conditions for the symmetry of the Y mode. Figure 1c compares the finite-difference numerical derivatives to the result given by the perturbation theory of eq 2 for both the resonant wavelength and the quality factor of the QNM of interest, which show clear quantitative agreement. We observe that, for small values of *S*_1*x*_, the displacement leads to a red shift, as expected from the increased effective refractive index, and to an increase of *Q*, as evidenced earlier in ref (19). By mapping out the real (Δω) and imaginary (Δγ) parts of the integrand of eq 2 for a displacement set **S** = {(*S*_*ix*_, 0)|*i* ∈ [1, *N*]} in the unaltered L3 cavity (*S*_1*x*_ = 0 nm), as shown in Figure 1d, it also becomes apparent that most holes around the cavity region produce considerable changes simultaneously to the loss rate γ and the frequency ω, warranting automated optimization of *Q* with respect to the position of all holes. In the following section, we report on the gradient-descent optimization of photonic cavities, where the objective function is the quality factor *Q* of a single QNM and where eq 2 is used to estimate the gradients relative to the in-plane position of all holes (see Supplementary Section S1 for details). Although the literature on optimal line search methods is vast, we employ here a simple line search direction along the gradient and a step length set to η∇_**S**_*Q*/|∇_**S**_*Q*|, with η chosen to produce sufficiently smooth convergence (see Supplementary Section S2 for a study on the effect of η). We note that no constraints are imposed on the performed optimizations, although inequality constraints to limit wavelength excursions can be readily implemented with the real part of eq 2 and additional constraints might be incorporated with adjoint-based sensitivity analysis.

## Results and Discussion

**Figure 2:**
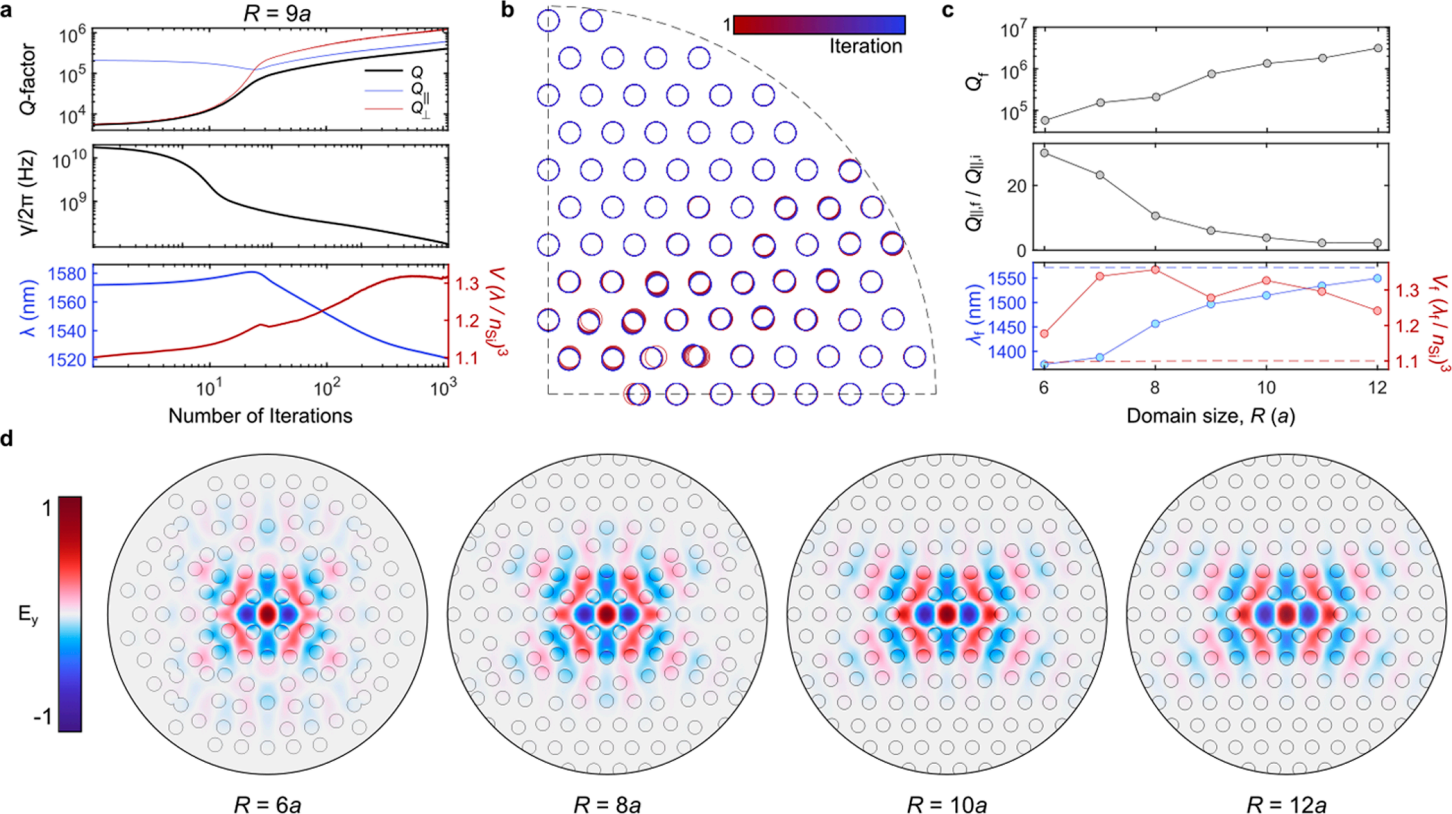
**Optimization of***Q***for L3 photonic-crystal cavities of varying footprints**. The *Q*-factor of L3 cavities is optimized by tuning all hole positions within a circle of radius *R*. (a) Evolution of the (top) quality factors *Q*, *Q*_∥_, and *Q*_⊥_, (middle) the loss rate, γ, of the QNM, and (bottom) the resonant wavelength and the mode volume, *V*, of the cavity mode. (b) Evolution of the position of all holes in the bottom-left quadrant of the photonic-crystal plane from the initial (red) to the final configuration (blue). (c) Dependence of cavity parameters with footprint *R*. From top to bottom: Optimized quality factor, *Q*_f_, ratio of the final to the initial *Q*_∥_, and final resonant wavelength λ_f_ and mode volume *V*. The blue and red dashed lines in the bottom panel indicate, respectively, the values of the initial wavelength and mode volumes for each *R*. (d) *y*-Component of the electric field, *E*_*y*_, in the plane *z* = 0 of the optimized L3 cavities with footprints *R = 6a*, *R* = 8*a*, *R* = 10*a*, and *R* = 12*a*. The fields are normalized to their maximum.

Most previous research on photonic-crystal slab cavities has focused on maximizing *Q*_⊥_ as *Q*_∥_ scales with the size of the etched pattern around the cavity defect, i.e., the number of Bragg mirrors. However, the optimization of *Q* for a mode in an ungapped system (see the case of a random system later) requires an optimization approach that can simultaneously address *Q*_⊥_ and *Q*_∥_. To evidence the versatility of the method proposed to optimize for both, we perform a systematic study of the L3 cavity studied in Figure 1 (*S*_1*x*_ = 0 nm) for varying footprints, gauged via the domain radius *R* (in units of *a*) within which circular holes are considered. Figure 2a,b summarize the results of the *Q*-factor optimization for *R* = 9*a*, including the evolution with iterations of *Q*, the loss rate γ, the resonant wavelength, the mode volume (calculated at the center of the cavity^44^), and the position of the holes (from red to blue in Figure 2b). The *Q* of the initial unoptimized L3 cavity is considerably limited by out-of-plane radiation as evidenced by the value of *Q*_∥_, obtained by integrating the radiated power over the slab thickness at the edge of the PML-backed domain,^63^ which is much higher than that of *Q*_⊥_. Therefore, an initial drop in *Q*_∥_ is observed, but both *Q*_∥_ and *Q*_⊥_ grow steadily after 20 iterations, indicating that the optimized configuration naturally accounts for both loss pathways, which for the final configuration in *R* = 9*a* are approximately of equal importance. We also observe that the minimum in *Q*_∥_ is accompanied by a maximum in the evolution of the resonant wavelength, for which we observe a 50 nm deviation between the initial resonant wavelength, λ_i_, and the final one, λ_f_. On the other hand, *V* slightly increases, but the 2-order-of-magnitude improvement in *Q* largely overcomes that uncontrolled increase in *V* in terms of the achieved Purcell factor. The associated position of the circular holes as iterations evidences that while the optimization displaces the holes bounding the defect, i.e., those considered in previous attempts to optimize the *Q* of this mode,^34^ the position of all holes along and around the 30.7° diagonal and up to the PML evolves during optimization. Such a direction nearly corresponds to that with the largest Bragg length in triangular lattice photonic crystals with circular holes, indicating that in-plane losses are, by construction, integral to the automated optimization strategy presented here.

We optimize the L3 cavities with different *R* using the same finite-element mesh sizes and fixed optimizer parameters, i.e., η = 2, and the stopping criterion to be the point when the relative variation between the *Q* of the running iteration and the *Q* 100 iterations before is less than 0.2%. Such a stopping criterion is used to account for the noisy nature of the evolution of *Q* as the number of iterations becomes large, which stems from the large value of η (see Supplementary Section S2). The effect of domain size on the optimized quality factor, *Q*_f_, which is shown in the top panel of Figure 2c for values of *R* varying from 6*a* to 12*a*, is pronounced. The transition from geometries limited by in-plane losses to those limited by out-of-plane losses is clear from an evaluation of the ratio *Q*_∥,f_/*Q*_∥,i_. Specifically, the large ratios for small *R* indicate that the dominating source of losses is in-plane losses, while the drop to 1 for large values of *R* indicates that the spatial extent of the photonic-crystal cladding already provides enough in-plane loss suppression and therefore the optimization is, in practice, optimizing *Q*_⊥_. As a consequence, this leads to only minor modifications around the defect for large *R* and produces only a small wavelength blue shift, as shown in the bottom panel of Figure 2c, where the final wavelength λ_f_ and mode volume *V*_f_ (solid-dotted lines) are compared to their initial values (dashed lines) for every value of *R*. We observe an increasing blueshift of λ_f_ relative to λ_i_ for decreasing *R*. We also report in Figure 2d the spatial profiles of the *y*-component of the electric field *E*_*y*_ of the optimized modes in the plane *z* = 0 as well as the position of the hole boundaries. While the final configuration of the holes can deviate considerably from the initial one, e.g., *R* = *6a* or *R* = 8*a*, the modal structure is preserved regardless of *R*. This stems from the fact that the boundary conditions determine field orientations on the symmetry axis and that the single QNM tracked is well-isolated spectrally and spatially.

**Figure 3:**
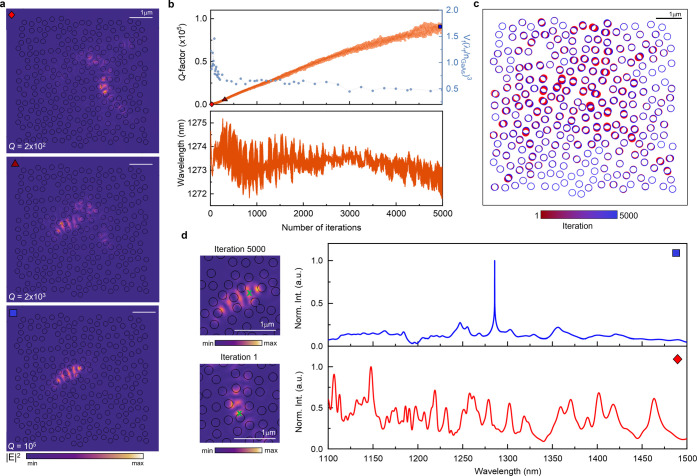
**Optimization of Q for a photonic mode in a random system.** Results of the optimization process applied to a random hole pattern in a slab of thickness *d* = 180 nm and with air holes of radius *r* = 110 nm. (a) Electric field intensity maps of the Anderson mode in the initial configuration (red diamond), in an intermediate step of the optimization process (dark-red triangle), and at the end of the optimization (blue square). (b) Evolution of the resonant wavelength (bottom panel) and quality factor and mode volume *V* (upper panel) of the Anderson mode. (c) Evolution of the position of all holes in the random design from the initial configuration (red) to the final one (blue). (d) FDTD spectrum of the initial random system (red spectrum) and at iteration 5000 (blue spectrum). The maps in the insets at the right of the spectra give the indication of the position of the dipole emitter (green crosses) used in the FDTD simulations.

On the contrary, random systems typically exhibit a large spatial and spectral density of (localized) modes in a given physical domain, which, for example, is used to alleviate issues in spectro-spatial matching to solid-state light emitters.^21^ Therefore, the implications of using the QNM perturbation theory to optimize the *Q* of a single QNM in a disordered system are far from obvious and can eventually lead to a strong variation in the mode structure, including its spatial location and confinement level, as we demonstrate here. We apply the optimization method to an Anderson mode supported by a gallium arsenide slab (*n*_GaAs_ = 3.46) of thickness *d* = 180 nm, size 36 μm^2^, and including *N* = 260 etched holes of radius *R* = 110 nm (see Supplementary Section S3 for details on the distribution of the position of the holes, e.g., the structure factor). The particular QNM we optimize, whose electric field intensity distribution is reported in the first map of Figure 3a, has an initial *Q* of 200, λ = 1273 nm and mode volume *V* = 1.22(λ/*n*_GaAs_)^3^, and is selected among the many other modes supported by the structure because it is spatially isolated from the rest and it is the highest *Q* in a close spectral neighborhood (see Supplementary Section 4 for visualization of other QNMs). The latter facilitates tracking of the QNM of interest as iterations evolve. The optimization process is run for 5000 iterations, and the evolution of *Q*, the resonant wavelength, and the hole positions are summarized in Figure 3b,c. The value of *Q* grows at a rather (average) constant pace and reaches *Q* = 10^5^ after 5000 iterations, which constitutes, to the best of our knowledge, the highest *Q* reported in a purely random system on a slab. We note that the steady increase in *Q* is also accompanied by considerable fluctuations, which originate because of a too large choice for η (η = 5) (see Supplementary Section 2). Fluctuations are also observed for the resonant wavelength of the mode although no significant drift is observed in this case. We attribute this to the random nature of the design that allows the holes to shift in any direction in the plane. Interestingly, monitoring how the spatial profile of the mode evolves as the *Q*-factor increases evidences that the mode location and spread evolve and therefore that the initial QNM chosen should be considered just as a seed for the optimization, contrary to the L3 cavity case. The three panels of Figure 3a highlight two specific configurations in addition to the initial one, corresponding, respectively, to *Q* = 2000 and *Q* = 10^5^. The middle configuration is chosen to highlight that the final one, despite the dramatic change in the spatial profile, is linked to the initial one, since the intermediate-case profile still preserves a tail corresponding to the original hotspot. The final optimized mode is located in a completely different position, and by tracking also the evolution of the mode volume *V* (light-blue dots in Figure 3b), we observe that it exhibits a much tighter localization (*V* = 0.4(λ/*n*_s_)^3^), leading to a *Q*/*V* = 5 × 10^6^ μm^–3^. This corresponds to an increase of the Purcell factor from 12 to 18 600, a final value typical of the best photonic-crystal cavities.^19^ Interestingly, the optimized configuration exhibits peculiar properties of both order and disorder; despite the uncorrelated disorder environment, a high-*Q* Anderson mode with a tight spatial localization (typical of point defects in a perfect photonic order) is displayed, in a system with a high spectral density of modes (typical of random photonic patterns). In order to numerically test the general validity of the optimization approach for random media, we apply the method to different initial QNMs of the same disordered system and to a photonic mode supported by a photonic crystal with a certain degree of disorder, i.e., based on a *quasi-ordered* distribution of holes. The results are shown in Supplementary Sections 5 and 6.

We evaluate the in-plane losses in the initial and optimized configuration and report that *Q*_∥_ increases from *Q*_∥_ = 1.3 × 10^5^ (unperturbed mode) until it reaches a value of *Q*_∥_ = 2.8 × 10^5^. This, similar to the case in Figure 2, demonstrates that in the initial configuration *Q* is strongly limited by the out-of-plane losses, which are then optimized at the end of the process, for which *Q*_∥_ ∼ *Q*. To understand the outcome of the optimization not only in terms of the single QNM we optimize but also in terms of the local density of optical states in the frequency range around it, we investigate the spectral response of the system in the presence of a single point-like electric dipole. To do this, we employ a finite difference time domain (FDTD) commercial software (Lumerical^64^) and use a spectrally broad (δλ = 200 nm and pulse length 7.28 fs) electric dipole located at the brightest spot of the explored QNM, as highlighted for initial and final configurations with a green cross in the zoomed-in field maps of Figure 3d. We report the spectrum of the structure for both the initial and final configurations in Figure 3d. The FDTD method confirms the stability of the mode central wavelength during the optimization process and the increase of the total *Q* by 3 orders of magnitude. Interestingly, the high density of modes typical of random systems prevails after the optimization as can be deduced from the presence of many other less prominent peaks in the emission spectrum. This evidences that the optimization of *Q* does not occur through the formation of a band gap as it is achieved in other disordered systems.^22−24^ This is further corroborated by the presence, in the final configuration, of other QNMs in spatial and spectral proximity (see Supplementary Figure S4 and S5) and by the very limited change to the hole statistics (see Supplementary Figure S3). The possibility of achieving a *Q*/*V* comparable to photonic-crystal cavities while preserving the high density of modes in a small spatial footprint might pave the way to the engineering of multiple Anderson modes in the same structure once the appropriate constraints are provided.

## Conclusions

In conclusion, we have proposed a gradient-based automated shape optimization approach to maximize the quality factor *Q* of optical resonances. The method, which employs first-order non-Hermitian quasinormal mode (QNM) perturbation theory for shape deformations, allows the efficient computation of the gradients of *Q* relative to small material boundary displacements without the need for solving additional (non)linear algebraic systems. Due to the free-form and boundary-conformal meshes employed in finite-element method simulations, the additional calculations are also trivial, making the actual calculation of the QNMs the only time- and memory-consuming step. Although the cases considered here are limited to hole displacements in dispersion-less and absorption-less dielectrics, the approach naturally extends to absorptive media^44,58^ and arbitrary—down to the mesh size—boundary deformations. We benchmarked our method with the optimization of cavity modes in dielectric slabs with either ordered or disordered patterns of scatterers. By simulating a standard L3 photonic-crystal cavity, we demonstrated that the approach can simultaneously take into account in-plane and out-of-plane losses and therefore truly optimize *Q* for a given domain size, circumventing issues found in other methods based on mode-expansion techniques.^34^ Such optimized low-footprint cavities may play a prominent role in applications where compactness determines functionality, such as spatial light modulators^65^ or electrically driven nanolasers,^66^ and enable optical interconnects for on-chip electronic–photonic integration, where size discrepancy has slowed down developments.^67^ While single QNM perturbation theories are more intuitively suited to systems with well-isolated QNMs, the method is also successfully employed on a random system with a large density of optical modes around the targeted initial QNM. We optimize the *Q* of an Anderson-localized mode, for which we obtain an increase of 3 orders of magnitude. The optimized mode also exhibits a decrease of the mode volume and an unchanged resonant wavelength, leading to a *Q*/*V* of 5 × 10^6^ μm^–3^, on par with photonic-crystal cavities.^19^ Our result might be relevant for the employment of random structures for lasing^68−70^ and sensing^71^ applications but also for the basic physical insights it can provide on light confinement in random systems. We foresee that the optimization approach in a random system of larger size might unveil novel features of engineered disordered systems such as hole structural correlations that are yet unexplored.

## Supporting Information Available

Details on the simulation models, the role of the gradient-descent η parameter, the statistical properties of the hole positions in the random systems, the QNMs of the initial and optimized random systems, and results of the optimization for different QNMs (PDF)
