# Correction to “Q-Factor Optimization of Modes in Ordered and Disordered Photonic Systems Using Non-Hermitian Perturbation Theory”

**DOI:** 10.1021/acsphotonics.4c00229

**Published:** 2024-02-16

**Authors:** Nicoletta Granchi, Francesca Intonti, Marian Florescu, Pedro David García, Massimo Gurioli, Guillermo Arregui

In the manuscript Introduction, where the role of short-range correlations on the quality factors of Anderson-localized optical modes is discussed, ref 24 [VascoJ. P.; HughesS.ACS Photonics2019, 6, 2926] was used inappropriately as the authors focus instead on long-range correlations. The correct reference is VascoJ. P.; HughesS.Phys. Rev. B2017, 95, 224202, which should be considered ref 24 in the corrected version of the manuscript.

The previous ref 24, which is now referred to as ref 24*, remains of outmost relevance for the manuscript as it demonstrates the important role of long-range correlations in the *Q*-factor of disorder-induced optical modes in slow light photonic-crystal waveguides, with inter-hole correlations leading to more than 2 orders of magnitude enhancement in the average *Q* compared to uncorrelated size disorder. Therefore, we highlight the following rephrasing in the introduction by adding ref 24*:

“Such an issue has been addressed at the ensemble-average level by introducing short-range correlations,^22–24^ but *Q*s of Anderson-localized modes are only on par with engineered cavities in the case of slow-light photonic crystal waveguides subjected to minute fabrication disorder,^25^ especially in the presence of long-range inter-hole correlations.^24*^”

