# Supplementary material for: Q-Factor Optimization of Modes in Ordered and Disordered Photonic Systems Using Non-Hermitian Perturbation Theory
Source: ACS Photonics. 2023 Jul 10;10(8):2808–15. doi: 10.1021/acsphotonics.3c00510 (PMC10436348; doi:10.1021/acsphotonics.3c00510)
Supplement: Supplementary file 1 — ph3c00510_si_001.pdf [file ph3c00510_si_001.pdf]

Supporting Information:

Q-factor optimization of modes in ordered and disordered photonic systems using non-Hermitian perturbation theory

Nicoletta Granchi,[1,2,*] Francesca Intonti,[1,2] Marian Florescu,[3]
Pedro David García,[4] Massimo Gurioli,[1,2] and Guillermo Arregui[5,*]

[1] *Department of Physics, University of Florence, via Sansone 1, I-50019 Sesto Fiorentino (FI), Italy*

[2] *European Laboratory for Nonlinear Spectroscopy, via Nello Carrara 1, I-50019, Sesto Fiorentino (FI), Italy*

[3] *Advanced Technology Institute and Department of Physics, University of Surrey, Guildford, Surrey GU2 7XH, UK*

[4] *Instituto de Ciencia de Materiales de Madrid (ICMM), Consejo Superior de Investigaciones Científicas (CSIC), Calle Sor Juana Inés de la Cruz 3, 28049 Madrid, Spain*

[5] *Department of Electrical and Photonics Engineering, DTU Electro, Technical University of Denmark, Building 343, DK-2800 Kgs. Lyngby, Denmark*

(Dated: July 7, 2023)

Supporting Information includes:

S1. Quasinormal mode (QNM) modelling and gradient estimation
S2. Role of the gradient-descent step η
S3. Statistical properties of disordered scatterers
S4. Evolution of the eigenstates through optimization
S5. The role of the initial QNM in a disordered system
S6. Optimization of a mode in a quasi-ordered system

S1. Quasinormal mode (QNM) modelling and gradient estimation

We simulate the QNMs of ordered and disordered systems using a finite-element method and a complex eigensolver based on the commercial software COMSOL Multiphysics. Figure S1 reports the geometry, including boundary conditions, and a characteristic mesh for an L3 cavity with $R = 6a$. Given the symmetry of the geometry and of the fundamental mode of the L3 cavity, we simulate an eighth of the cavity and impose a perfect magnetic conductor (PMC) bounary condition on the zy-plane (red) and xy-plane (green) and a perfect electric conductor (PEC) boundary condition on the zx-plane (blue). The radiation boundary condition characteristic of QNMs is emulated using a perfectly matched layer (PML), as highlighted in yellow. In the case of the disordered systems, only the symmetry relative to the slab mid-plane is preserved as is therefore the xy-plane PMC.

We mesh all geometries in this work (including the random structures) using tetrahedral mesh elements. Meshes are build by first setting a free triangular mesh with mesh-element size $h = 2\pi\mathrm{R}/18$ nm on the cylindrical hole boundaries and the rest, except for the PML, is meshed with a free tetrahedral mesh with a minimum element size of $h_{\mathrm{min}} = a/12$ nm, a maximum element size of $h_{\mathrm{max}} = a/4$ nm (with a lattice constant of the photonic crystal) and a maximum mesh element growth rate of 1.4. The PML uses a structured mesh with 5 elements along its width. Fig. S1b shows the resulting mesh for the optimized L3 cavity with $R = 6a$.

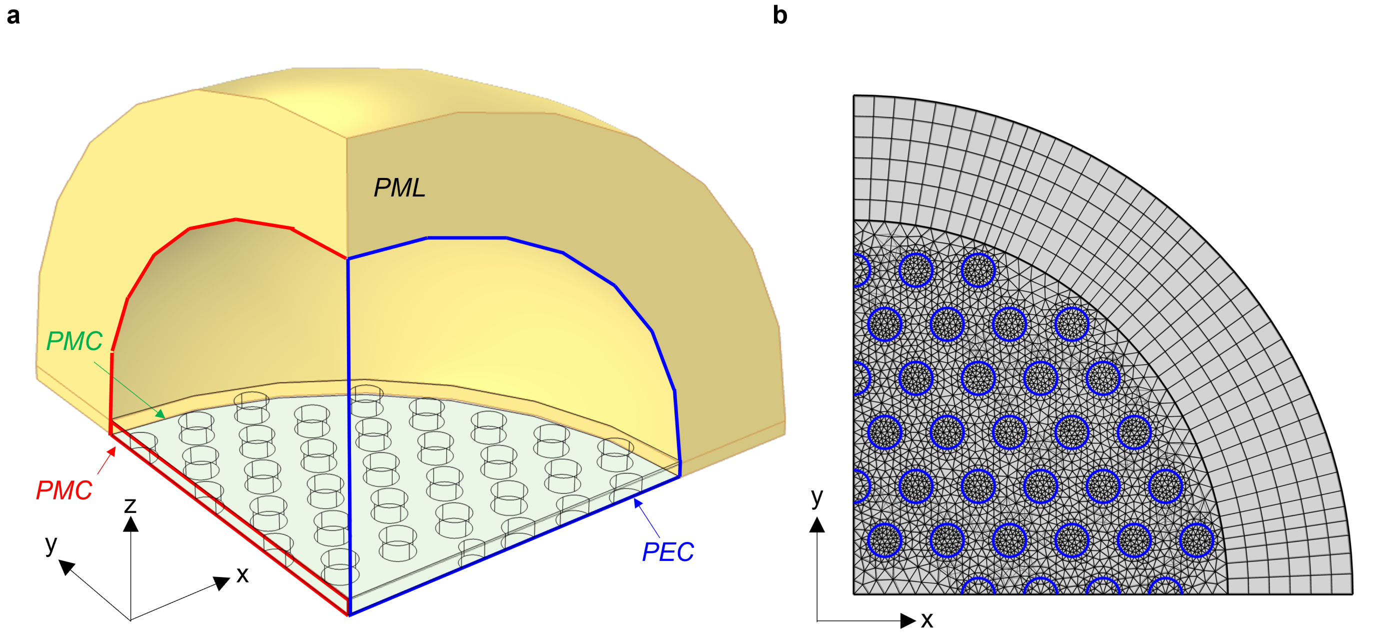

Supplementary Figure S1: Characteristic finite-element model for finding QNMs. (a) Sketch of the employed geometry to simulate an L3 cavity, where the boundary conditions are highlighted in different colors. (b) Rendering of the mesh used to solve an optimized L3.

We perform a complex eigenmode analysis and solve for the two QNMs with smallest distance (in absolute value) to a predefined reference frequency, ω_{ref}. The models are solved on a workstation with an Intel Core i9-10900X processor (10 core, 19,25 MB cache, 3,7 GHz, 4,7 GHz Turbo, HT, (165 W), DDR4 memory at 2.666 MHz, not ECC) and 128 GB of RAM and take from 2 min for the smallest L3 cavity to around 30 min for the largest random system. Once the eigenmode of interest, $\{\omega_n, \gamma_n, \mathbf{E}_n(\mathbf{r})\}$, is selected, the gradient of Q is computed as follows:

1. The considered displacements are set to be $\mathbf{s}(\mathbf{r}) = (S_{ix}, 0)$, with i the index for the *i-th* hole.
2. Eq. 2 is integrated in the boundary of the *i-th* hole by using numerical integration schemes, with the Gauss points of the elements used for the integration. With this, we find $\frac{\partial\omega_{\mathrm{n}}}{\partial s_{ix}} = \mathfrak{Re}(\Delta\tilde{\omega}_{\mathrm{n}})/S_{ix}$ and $\frac{\partial\gamma_{\mathrm{n}}}{\partial s_{ix}} = \mathfrak{Im}(\Delta\tilde{\omega}_{\mathrm{n}})/S_{ix}$.
3. With those two in hand, the chain rule is used to find $\frac{Q}{\partial s_{ix}}$, i.e., $\frac{\partial Q}{\partial s_{ix}} = \left(\frac{\partial\omega_{\mathrm{n}}}{\partial s_{ix}}\gamma_{\mathrm{n}} - \frac{\partial\gamma_{\mathrm{n}}}{\partial s_{ix}}\omega_{\mathrm{n}}\right)/2\gamma_{\mathrm{n}}^2$.
4. This process is repeated (1 to 3) for all holes $i \in [1, N]$ and for vertical displacements $\mathbf{s}(\mathbf{r}) = (0, S_{iy})$.
5. All the obtained values are used to build $\nabla_{\mathbf{s}} Q$.

Once the gradient is obtained, we select the line search direction along the normalized gradient and set the hole displacements to $\delta\mathbf{P}_n = \eta\nabla_{\mathbf{s}}Q/|\nabla_{\mathbf{s}}Q|$. Then, we update all positions as $\mathbf{P}_{n+1} = \mathbf{P}_n + \delta\mathbf{P}_n$. In addition to that, the reference frequency for the eigensolver is adapted to $\omega_{\mathrm{ref}} = \omega_{\mathrm{n}} + \nabla_{\mathbf{S}}\omega_{\mathrm{n}} \cdot \delta\mathbf{P}_n$, which ensures that the next iteration the QNM of interest is found by the solver. In cases of very large valueS of η, e.g. $\eta = 16$ in Fig. S2, the number of modes found is extended to four, which extends the computation time slightly.

S2. Role of the gradient-descent step η

As it happens in any fixed-step gradient-descent method, the optimization path depends on the amplitude of the steps taken, i.e., the value of η. The values in the main text have been selected to guarantee a steady on-average growth of the Q-factor despite the presence of fluctuations. For completeness, we report here (Fig. S2) on the role of the parameter η for a low-footprint ($R = 6a$) L3 cavity, where the computation time for a single iteration is short enough to make such an analysis possible. The figure evidences that larger values of η can lead to faster optimization rates, but they also enhance the fluctuations we observe. Remark that the type of fluctuations observed also depends on η, e.g. for $\eta = 4$ they are very steady and for $\eta = 8$ or 16 they are large at

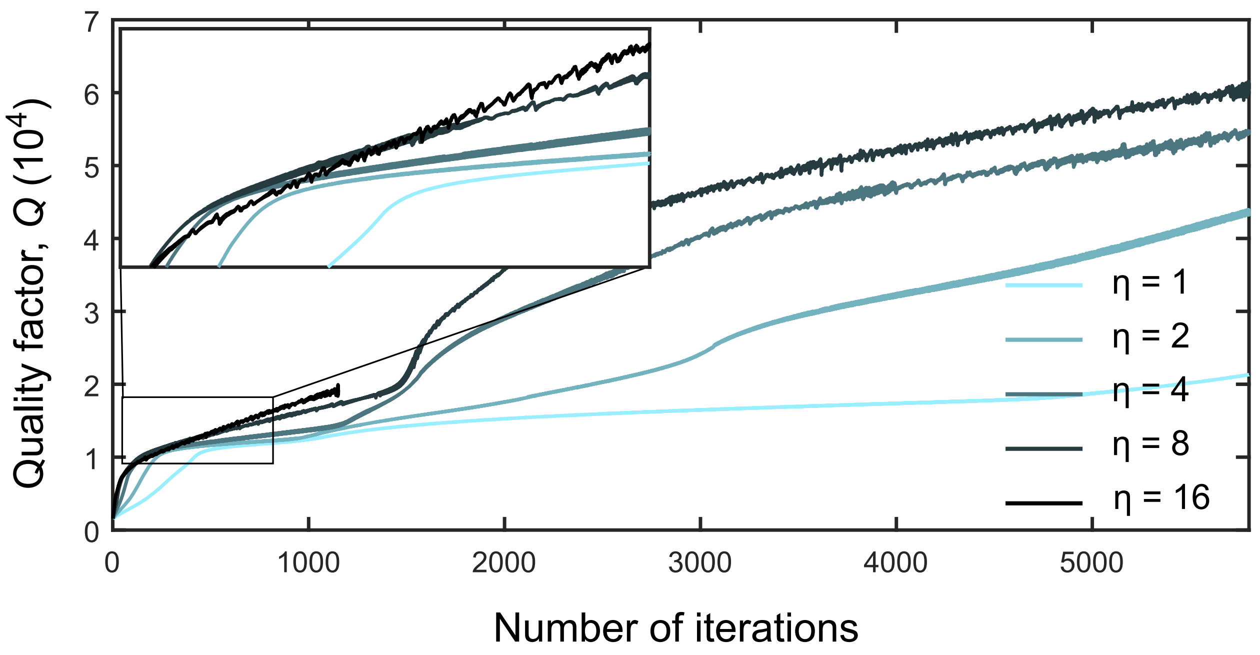

Supplementary Figure S2: **Effect of the gradient descent rate** η**.** Evolution of the quality factor Q of a low-footprint (R=6a) L3 photonic-crystal cavity with the number of iterations for varying value of eta. The inset shows the detailed region between iterations 20 and 820.

moments and nearly in-existent at others (see inset in Fig. S2). This likely indicates that the optimization landscapes changes depending on η and that the eventual optimum reached will only be local.

S3. Statistical properties of disordered scatterers

An important characteristic when studying wave systems made of distributed point-like particles is their spatial ordering, which can, under certain conditions, reveal features of the expected scattering properties. In Fig. S3a (b) we report on the analysis of the statistical distribution of holes in the initial (final) disordered geometries used in Fig. 3 in the main text. The position of the holes corresponding to the configuration of the un-optimized and optimized Anderson modes are shown in the first panel of Fig. S3a and b respectively. We calculate the autocorrelation function $g(\mathbf{r})$ [arXiv:2106.13892v1, (2021)] and its radial average $g_2(r)$ (second and third panel of Fig. S3a and b) which can be used to infer some statistical properties of the dielectric distribution. For example, the first x-axis intercept is associated with the average side of the inclusions (mediated by their random distribution) and the position of the first maximum r_{av} tells about the average separation between these inclusions. We obtain r_{av} = 144.28 nm and r_{av} = 142.88 nm respectively for initial and final configurations. The fourth and fifth panels in both Fig. S3a and b show the corresponding structure factors, $S(k)$, to which we have applied a zero-padding method to increase the resolution, and their radial average curves [Adv.Opt.Mat.,2102565,(2022)]. From the last we can deduce that although

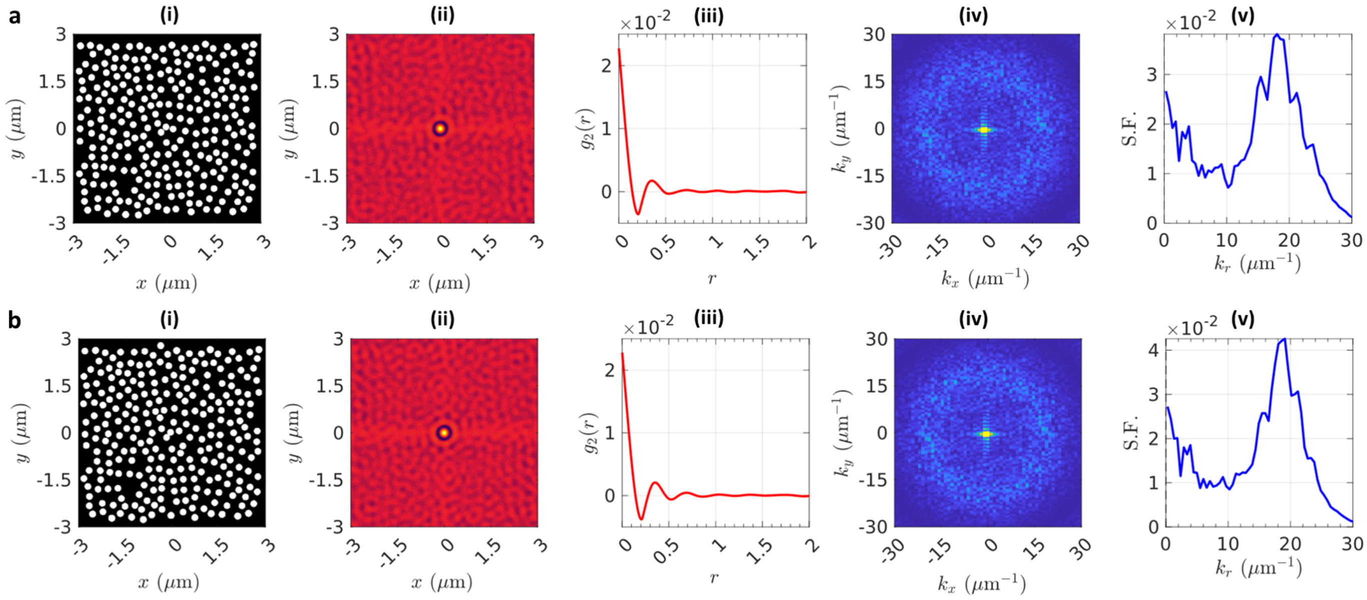

Supplementary Figure S3: **Evolution of the hole distribution statistics through optimization.** Calculations of the statistical distribution of holes in the initial (a) and final (b) disordered configuration. Panels (i): sketch of the holes position. Panels (ii) and (iii): autocorrelation function and its radial average $g_2(r)$. Panels (iv) and (v): Structure factor and its radial average.

the studied design belongs to the class of *random sequential addition* (RSA) patterns, which is not hyperuniform, both patterns present "emergent" short range order, as visible from the well-defined peak in $g_2(r)$ and the "emerging" strong scattering ring in $S(k)$.

S4. Evolution of the eigenstates through optimization

One of the key features of photonic crystals are photonic band gaps, where the density of states is depleted. Photonic-crystal cavities rely on point-like symmetry-based defects in those crystals to exhibit cavity modes with tight spectral and spatial localization and isolated from all other modes. However, gaps are not recurrently present in random systems, except for hole distributions with particular statistics. Therefore, an Anderson-localized mode is, in general, spectrally and spatially close to other modes, which might have an influence on the optimization path.In this section we report the modes (wavelengths, Qs and field profiles) found for the initial (Fig. S4) and final (Fig. S5) geometries of the optimization reported in Fig. 3 in the main text. The initial and final QNMs are highlighted in blue and red respectively. Interestingly, it would appear as if the local density of states around the cavity region had been partially depleted, i.e. the closest modes are not only further from the final QNM, but also at spatial locations with little overlap. However, it appears impossible to deduce whether the optimizer is depleting the density of states from a single optimization run.

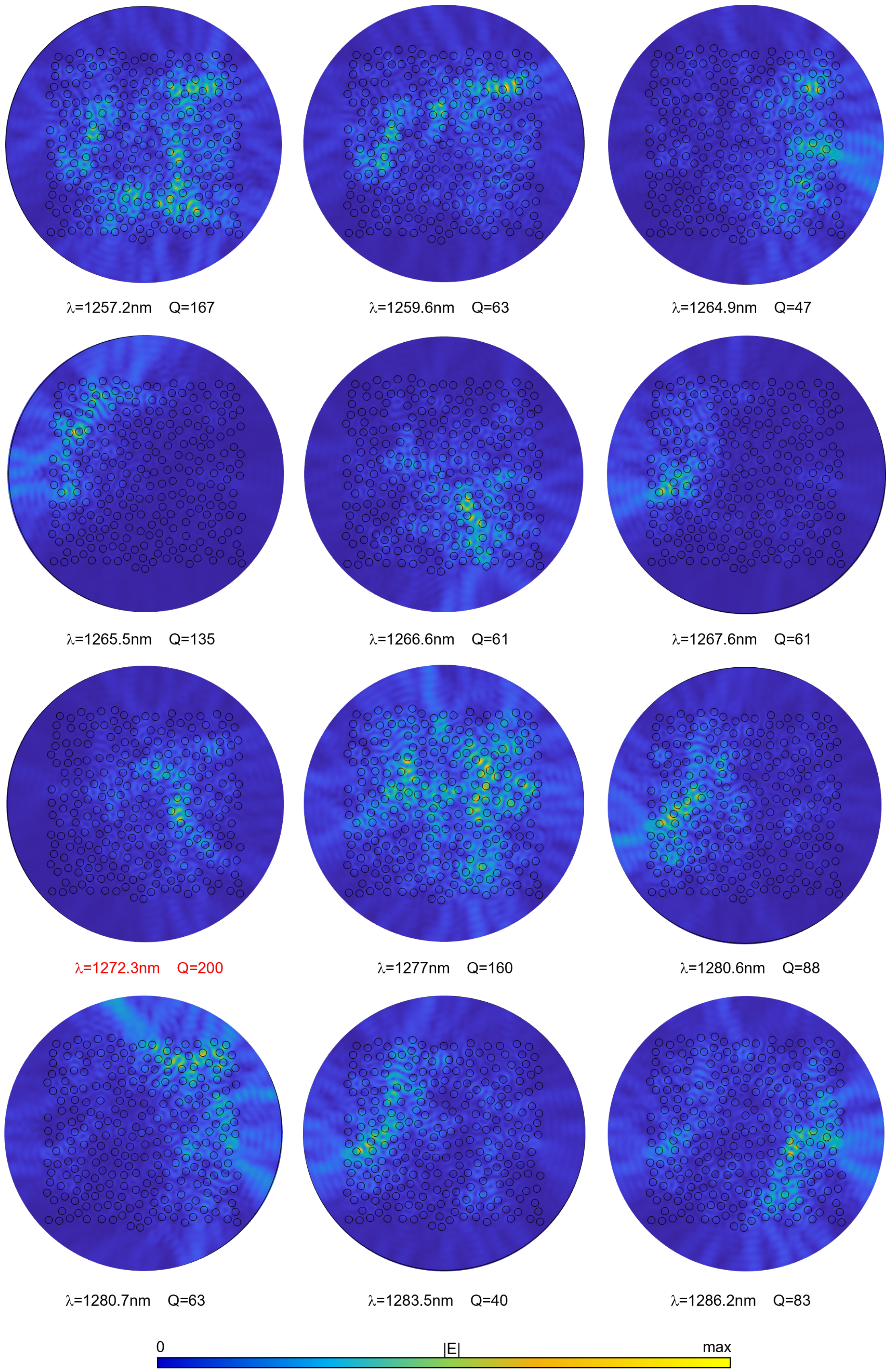

Supplementary Figure S4: **Electric field distributions of modes in the initial random configuration.** The mode chosen for the optimization reported in Fig. 3 in the main text is highlighted in red.

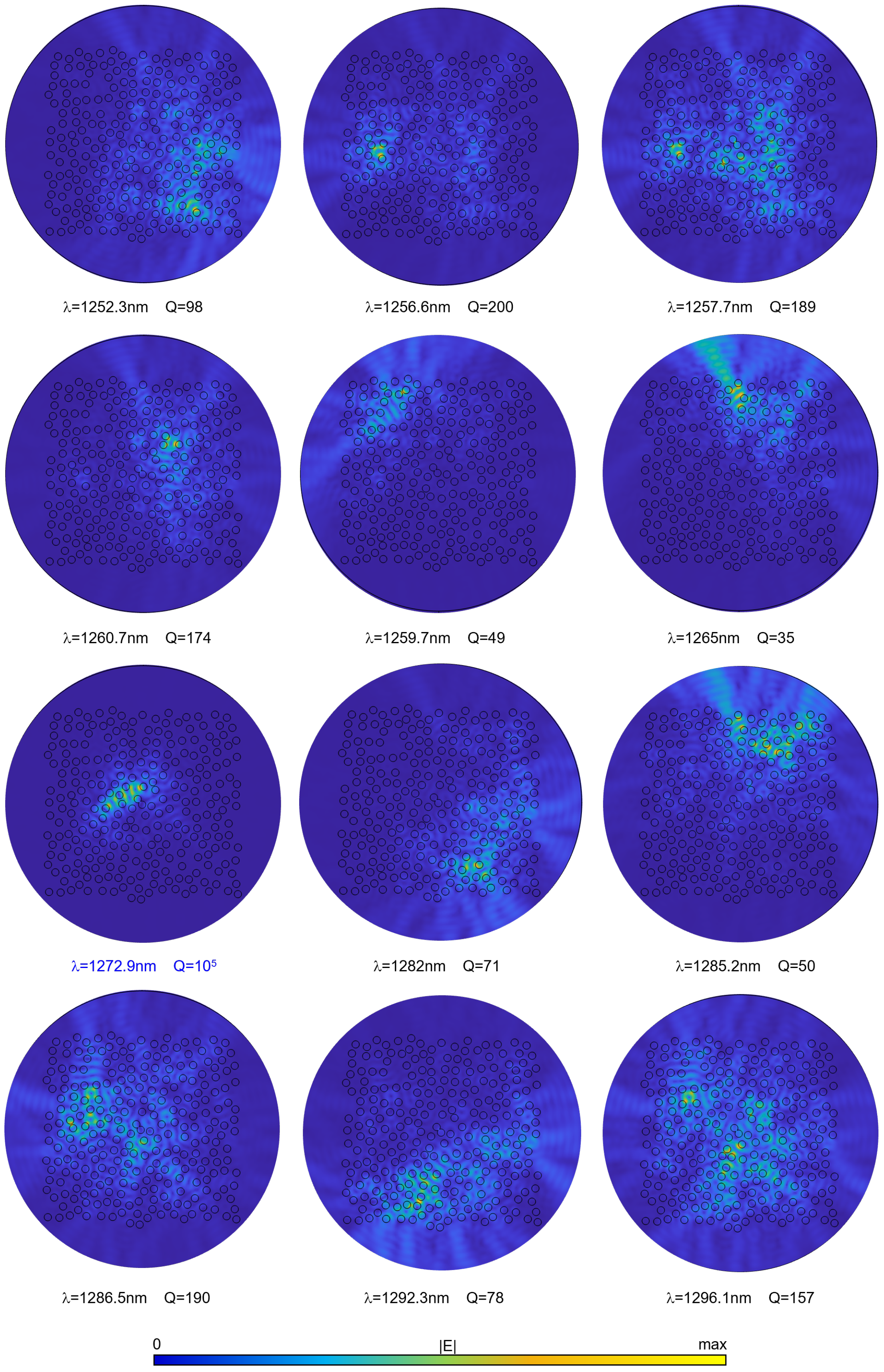

Supplementary Figure S5: **Electric field distributions of modes in the final random configuration.** The optimized mode chosen for the optimization reported in Fig. 3 in the main text is highlighted in blue.

S5. The role of the initial QNM in a disordered system

In the optimization of the Q-factor of a QNM in the disordered system of Fig. 3 in the main text, we observe that the optimized QNM has a completely different spatial distribution with respect to the initial one. This indicates that the initial QNM needs to be understood as a seed for the optimization, i.e. simply as an initial guess. This differs considerably from the case of the L3 cavity, where the mode structure is preserved and one can claim that, after optimization, it remains the fundamental mode of an L3 cavity. To better understand this aspect, we perform a limited number of iterations (compared to Fig. 3 in the main text) starting from two additional initial QNMs, namely those at λ = 1277 nm and at λ = 1266.6 nm (as shown in Fig. S4). The optimization results for

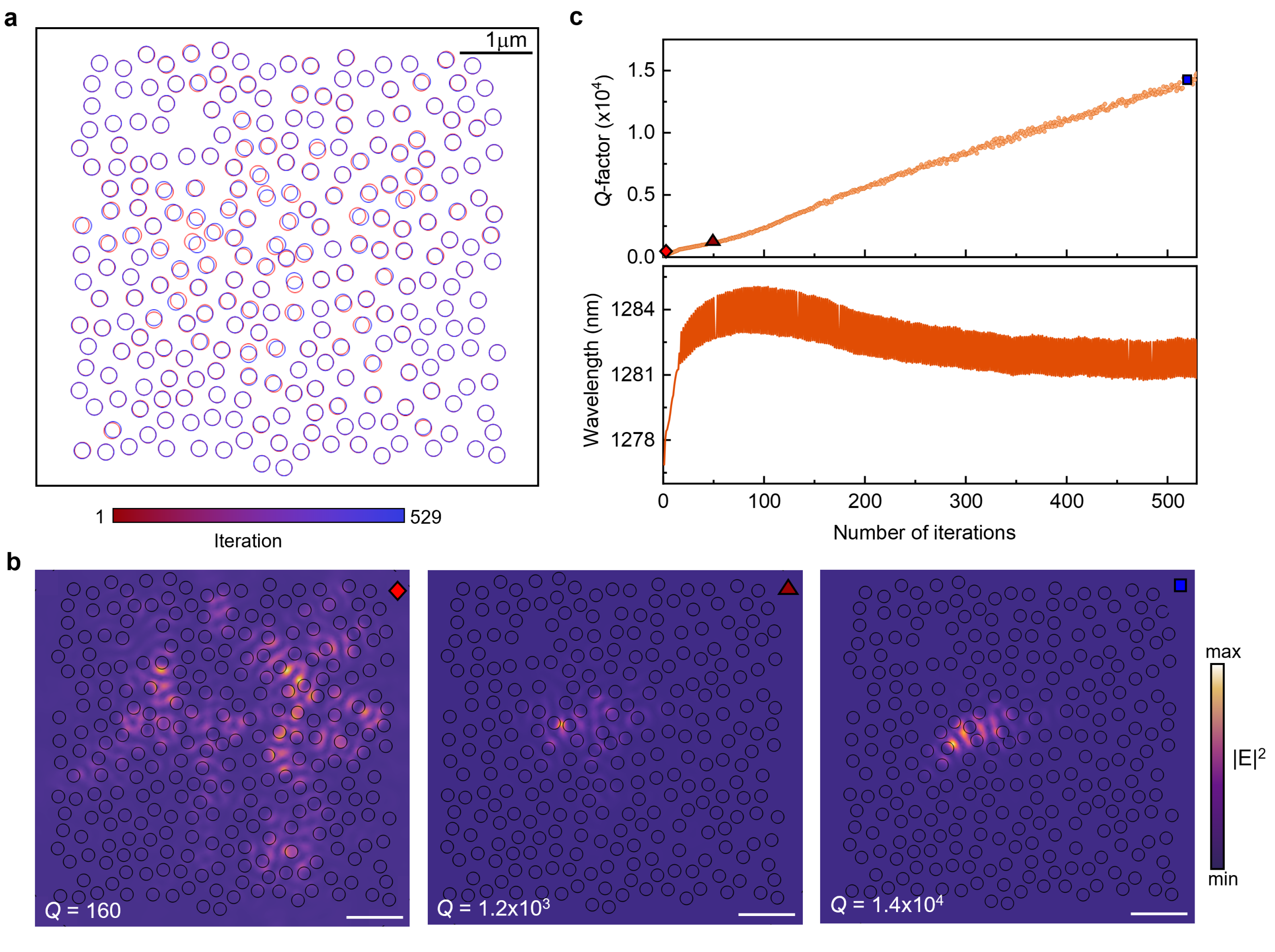

Supplementary Figure S6: Results of the optimization applied to another Anderson mode in the random design at λ = 1277 nm. (a) Evolution of the position of all holes in the random design from the initial configuration (red) to the final one (blue). (b) Electric field intensity maps of the Anderson mode in the initial configuration (blue diamond), in an intermediate step of the optimization process (dark-red triangle) and at the end of the optimization (red square). (c) Evolution of the resonant wavelength (bottom panel) and quality factor (upper panel) of the optimizing Anderson mode.

the first of the two other initial QNMs are reported in Fig. S6, where the value of η is fixed at 2. As expected from the large spatial extent of the initial QNM, we see that most of the holes in the random pattern have had their position modified (Fig. S6a). These modifications are accompanied by a steady evolution of Q, with an increase from 160 to 1.4x10^4 after 530 iterations. Interestingly, even if the employed QNM differs considerably form that of Fig. 3 in the main text, we see that after only 500 iterations the energy density reported in the right panel of Fig. S6b closely resembles that of the optimized mode in Fig. 3 in the main text. Its Q is still an order of magnitude below the final Q in the main text and the difference in resonant wavelengths is actually larger than the initial. However, we foresee that extending the number of iterations will likely make both optimizations converge towards the same local optimum. This is probably due to the very similar spatial distribution over the region where this particular mode overlaps with the one optimized in the main text and because it is the mode that is closer in wavelength. We therefore explored a second QNM, with very different spatial features with respect to the mode optimized in the main text. We also note that the overall growth rate of Q is very similar among the two optimizations, $\Delta Q/\Delta i \sim 25$, except for the first 10 to 15 iterations. Given that both use the same value of η it may also be an indication that both are roughly following the same optimization pathway. This is also confirmed by looking at the distribution of holes in real space, which seems to overlap relatively well despite the difference in the number of iterations.

As mentioned above, to further explore this aspect, we optimize starting from a third mode with a field profile that doesn't overlap spatially with that of Fig. 3 in the main text. The results of a preliminary optimization using such mode at $\lambda = 1266.6$ nm as the initial QNM are reported in Fig.S7 with analogue panels as the previous figure. The steady evolution of Q, with an increase from 134 to 2x10^3 after 135 iterations, confirms a similar growth rate for Q, accompanied by a tighter spatial localization. Interestingly, the spatial profile of the mode, initially located near the lower border of the pattern, drifts towards the center of the structure in order to decrease the losses. These results contribute to validate our Q-factor optimization procedure. In particular, when applied to Anderson modes that have a considerable initial extent, we see that it not only optimizes Q, but generally tends to decrease also V, leading to very large improvements in the Purcell factor.

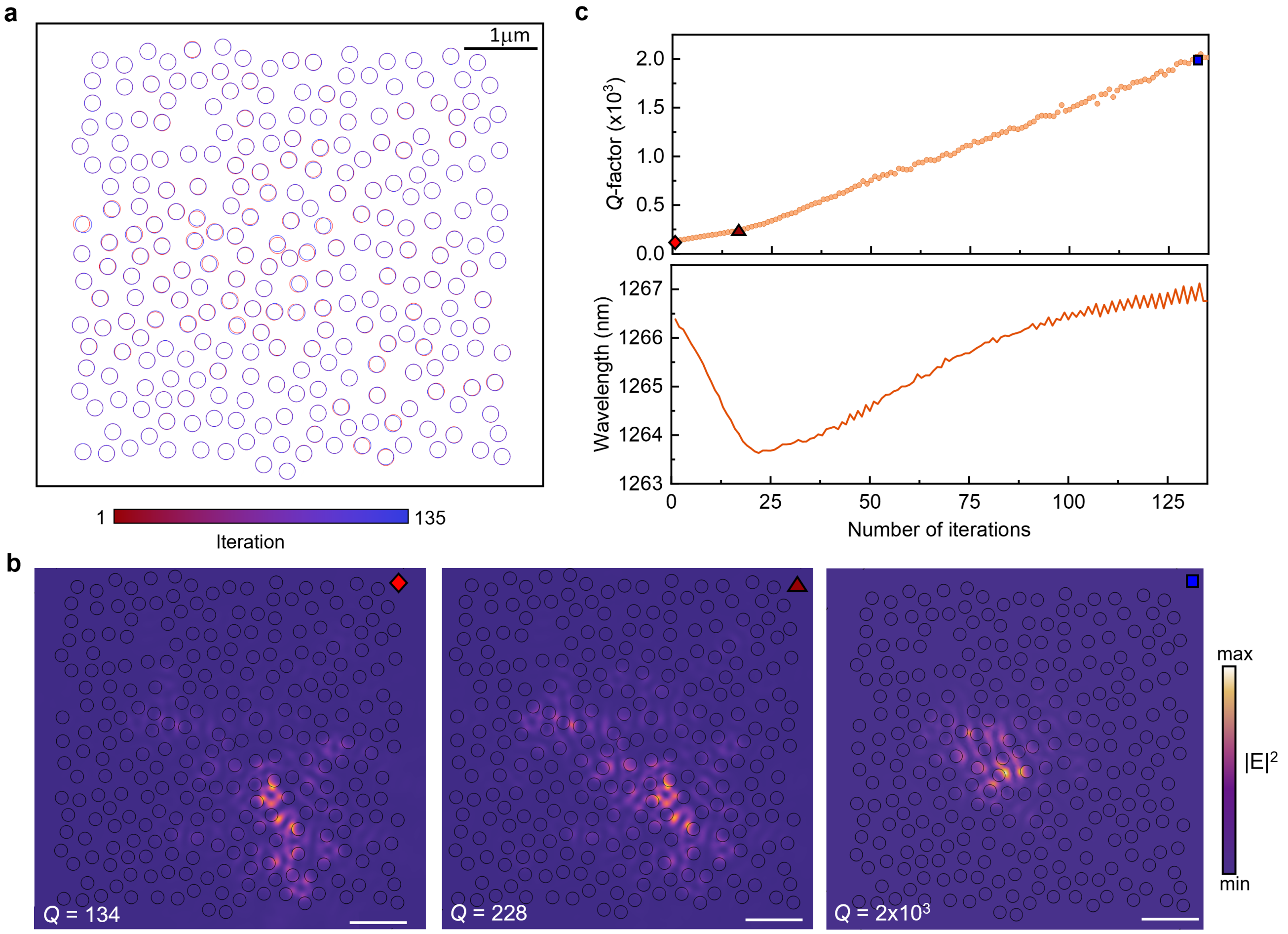

Supplementary Figure S7: Results of the optimization applied to another Anderson mode in the random design at $\lambda = 1266.6$ nm. (a) Evolution of the position of all holes in the random design from the initial configuration (red) to the final one (blue). (b) Electric field intensity maps of the Anderson mode in the initial configuration (blue diamond), in an intermediate step of the optimization process (dark-red triangle) and at the end of the optimization (red square). (c) Evolution of the resonant wavelength (bottom panel) and quality factor (upper panel) of the optimizing Anderson mode.

S6. Optimization of a mode in a quasi-ordered system

In this section, we report the preliminary results obtained by applying the same optimization method to a mode localized in a quasi-ordered photonic structure, as a proof of principle on the versatility of the approach. The structure considered here is based on a triangular-lattice photonic crystal (lattice constant $a = 320$ nm, holes radius $r = 90$nm and slab thickness $t = 180$ nm) where positional disorder is introduced by displacing the hole centers by $(\delta x_i, \delta y_i)$ following uncorrelated normally distributed random variables, e.g. $\delta x_i \sim \mathcal{N}(\mu = 0, \sigma)$. In Fig. S8 we report the optimization results after 200 iterations for a structure generated using $\sigma = 0.1a$. The initial geometry is shown in

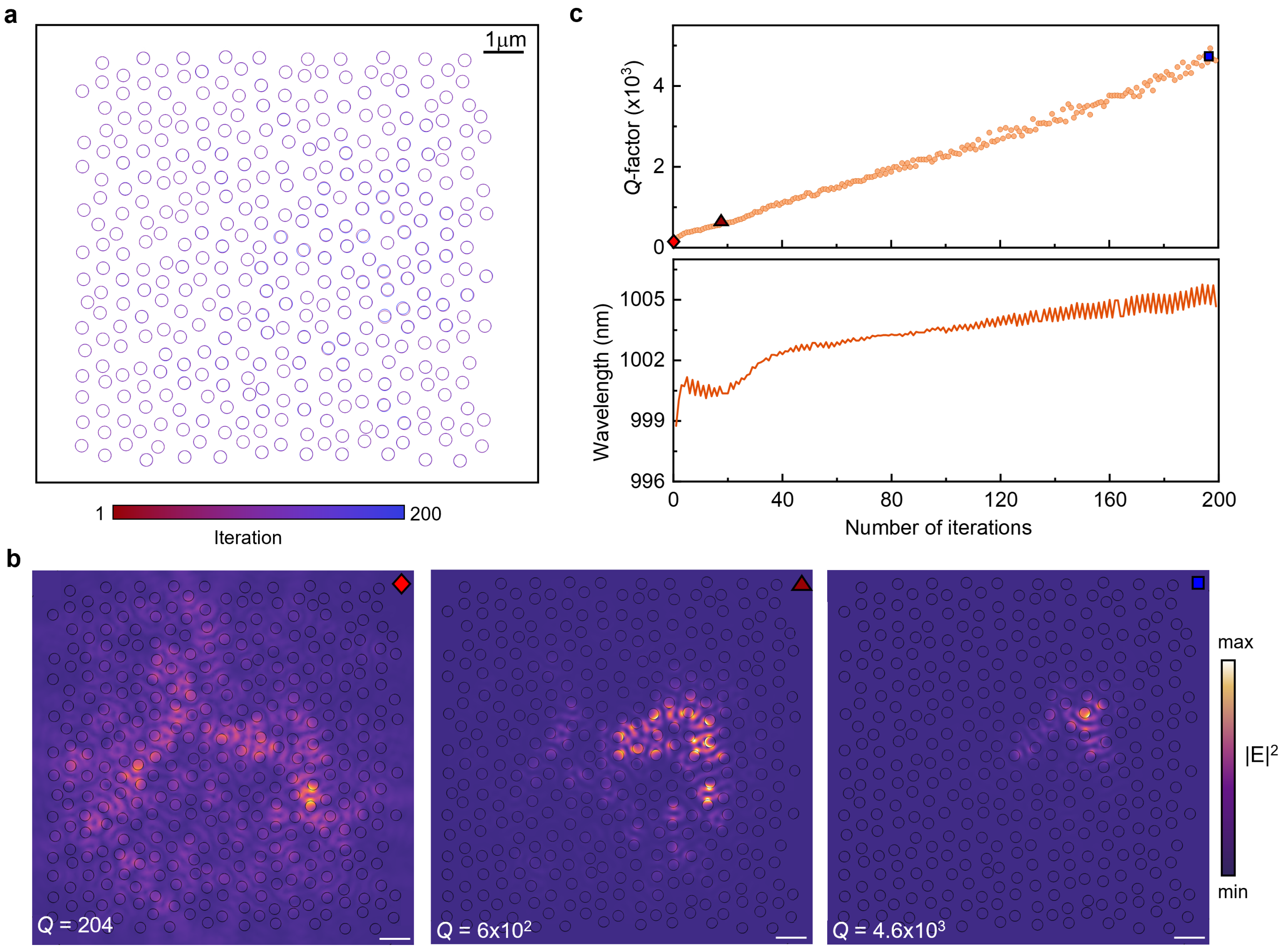

Supplementary Figure S8: **Results of the optimization process applied to a mode supported by a quasi-ordered system.** (a) Evolution of the position of all holes in the random design from the initial configuration (blue) to the final one (red). (b) EM Electric field intensity maps of the Anderson mode in the initial configuration (blue diamond), in an intermediate step of the optimization process (dark-red triangle) and at the end of the optimization (red square). (c) Evolution of the resonant wavelength (bottom panel) and quality factor (upper panel) of the optimizing Anderson mode.

blue in Fig S8a and the electric field intensity distribution of the initial QNM selected for optimization (λ=999 nm and Q=204) is shown in the first panel of Fig S8b and exhibits a fairly delocalized mode profile. The mode chosen is close in wavelength to the minimum of the air band of the underlying photonic crystal (at 1008 nm), and is therefore just at the edge of the Lifshitz tail of localized modes that disorder creates at the edge. By following the evolution of the resonant wavelength and quality factor (Fig S8c) we observe that in the first 200 iterations, the Q factor increases up to 4600, while the resonant wavelength slightly changes from 999 nm to 1005 nm. We attribute this progressive drift to higher wavelengths to the tendency of the mode to approach the in-plane gap of the underlying photonic crystal. By monitoring the spatial distribution of the modes, analogously to what is obtained for the random structure shown in the main text, we observe that the middle configuration (dark-red triangle) indicates that the final one, despite the dramatic change in the spatial profile, is linked to the initial one, since the intermediate-case profile still preserves a tail corresponding to the original hotspot. We also report a decrease in the modal volume from $V = 2(\lambda/n_{\mathrm{GaAs}})^3$ to $V = 0.98(\lambda/n_{\mathrm{GaAs}})^3$.

---

* Corresponding authors: granchi@lens.unifi.it, guibra@dtu.dk
